# Can Pulse Rate Variability be Used to Monitor Compliance with a Breath Pacer?

**DOI:** 10.1007/s10484-023-09617-y

**Published:** 2024-01-12

**Authors:** Sergey Sokolovskiy, Dahyana Arroyo, Paul Hansma

**Affiliations:** University of California, Santa Barbara, USA

**Keywords:** Pulse rate variability, Slow paced breathing, Compliance with breath pacer, Respiratory sinus arrhythmia, Dynamic phase extraction, Meditation

## Abstract

Slow paced breathing has been demonstrated to provide significant health benefits for a person’s health, and, during breathing sessions, it is desirable to monitor that a person is actually compliant with the breath pacer. We explore the potential use of pulse rate variability to monitor compliance with a breath pacer during meditation sessions. The study involved 6 human subjects each participating in 2–3 trials, where they are asked to follow or not to follow the breath pacer, where we collected data on how the magnitude of pulse rate variability changed. Two methods, logistic regression and a running standard deviation technique, were developed to detect non-compliance with the breath pacer based on pulse rate variability metrics. Results indicate that using pulse rate variability alone may not reliably detect non-compliance with the breath pacer. Both models exhibited limitations in terms of false positives and false negatives, with accuracy ranging from 67 to 65%. Existing methods involving visual, audio, and motion signals currently perform better for monitoring compliance with the breath pacer.

## Introduction

Slow paced-breathing has recently gained increasing recognition for its potential to improve an individual’s health and overall well being. Specifically, it has been shown that such breathing can significantly lower the blood pressure (Schein et al., 2001; Grossman et al., 2001; Rosenthal et al., 2001; van Hateren et al., 2014; Elliot et al., 2004; Meles et al., 2004; Landman et al., 2014; Schein et al., 2009; Altena et al., 2009), lower neural sympathetic activity (Oneda el at., 2010; Harada et al., 2014; Barros et al., 2014), decrease peripheral resistance (Gavish et al., 2011; Ovadia-Blechman et al., 2017; Faconti et al., 2019; Bachler et al., 2020) and arterial stiffness (Parati et al., 2008; Ekman et al., 2011; Bilo et al., 2012; Debicka-Dabrowska et al., 2015) as well as reduce anxiety and stress (Morarend et al., 2011; Ouseph et al., 2014; Gabriely et al., 2020). Slow paced breathing has also been utilized for Heart Rate Variability Biofeedback (HRVB) which involves training individuals to control their heart rate using slow breathing patterns (Lehrer et al., 2014). There has been innovative work done that shows benefits and positive effects of HRVB for one’s health and overall well being (Aschbacher et al., 2023; Lehrer et al., 2020; Goessl et al., 2017). There are many devices that can guide slow breathing (Miri et al., 2020; Martin et al., 2014; Kirby et al., 2009). However, it is important to monitor that the user is actually following the guided breathing pattern (Benetazzo et al., 2014), as it is common for people to get distracted and deviate from the breathing rate of the breath pacer.

In this light, several existing techniques, that used image (Nam et al., 2016a), sound (Nam et al., 2016b; Avalur, 2013) or motion recognition (Hernandez et al., 2015; Shen et al., 2017; Prigent et al., 2021), have been used in order to determine the breathing rate of the person. Specifically, such techniques as extracting breathing rate from image recognition (Benetazzo et al., 2014, Nam et al., 2016a) have demonstrated outstanding performance. For example, there exists an FDA-approved device, RESPeRATE that can detect a person’s breathing rate using a chest strap (Anderson et al., 2009). Such data then can be used to judge if the person’s breathing is compliant with the breath pacer during a meditation session (Zhang et al., 2013).

**Fig. 1:**
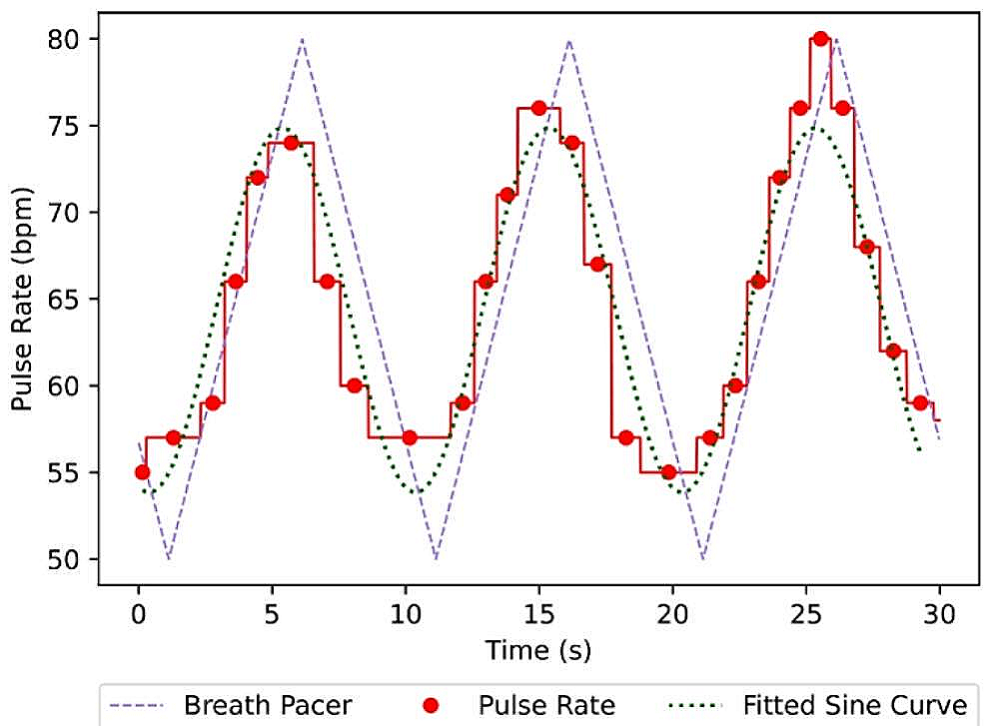
Variation in the pulse rate (PR) due to Respiratory Sinus Arrhythmia (RSA). When the person is following the breath pacer (dashed line), variation in the pulse rate closely follows that breathing pattern

In this paper we explored the possibility of using pulse rate variability to determine compliance with a breath pacer. Recently, a novel technique to calculate the magnitude of pulse rate variability has been developed known as Dynamic Phase Extraction (DPE) (Li et al., 2022). DPE calculates how much the pulse rate of a person varies during a single breath due to respiratory sinus arrhythmia as well as its phase shift relative to breath (Fig. 1). The magnitude drops significantly (Fig. 3a) as a person stops following the breath pacer and starts breathing at their own rate. Therefore, in this study we explored the possibility of using Dynamic Phase Extraction and change in magnitude of the pulse rate variability in order to determine the compliance with the breath pacer.

## Experimental Methods

**Fig. 2:**
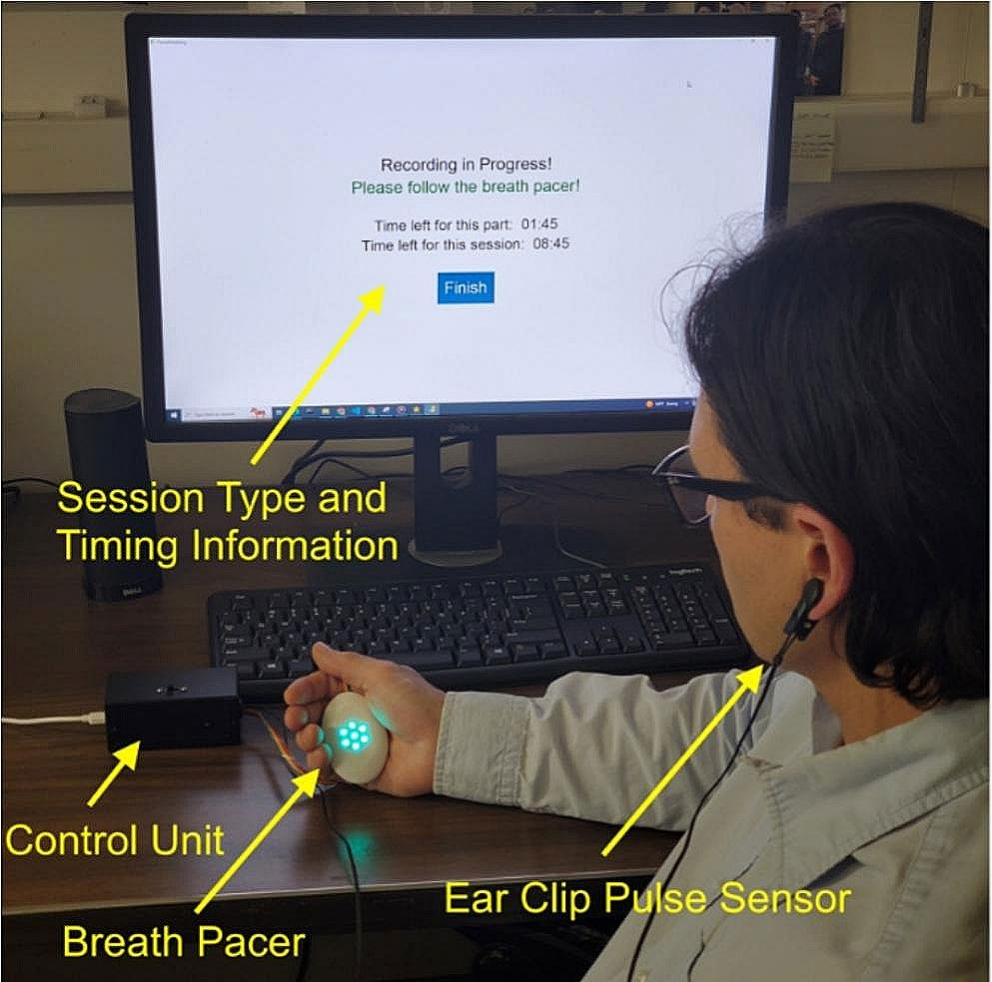
Experimental setup including device (control unit) with breath pacer and ear clip pulse sensor, and computer for data collection. Participant is seated in front of the computer in a padded chair and is wearing an ear clip pulse sensor. Participant is following a visual breath pacer and is guided to breathe according to the instructions on the computer screen with a timer

The device (control unit) (Fig. 2) that was used in the study consisted of a WEMOS LOLIN 32 board, Seeed Studio earclip pulse sensor, and a WCMCU-2812-7 board with 6 LED lights mounted on it. The WEMOS LOLIN 32 board was put in a metal case. Three wires were coming out of the case: one to power and read the signal from the pulse sensor, one to power and control breath pacer, and a micro USB cable that was connected to the desktop computer to power the device and collect the data from it.

The LED board was put in a white 3D printed oval shell, and attached to its inner surface such that the brightness of the light would be distinguishable through the shell. The breath pace was set using the brightness of the LEDs: increasing brightness meant inhale, and decreasing brightness meant exhale. The breath length was set to 10 s during all trials in the study.

Using collected pulse data and Dynamic Phase Extraction (DPE) Algorithm, the device computed the magnitude and phase of the pulse rate variability (PRV). Computed magnitude and phase along with timestamps of the breath were sent to the desktop computer with a serial communication. It received data using a Python Program, and saved it. This program also provided a graphical user interface that displayed directions on whether the subject had to or did not have to follow the breath pacer.

The study procedure was approved for use by the Human Subjects Committee through the University of California, Santa Barbara. The study was conducted throughout a one week period of 3 sessions on 3 different days of the week. Six participants (3 male and 3 female) of ages 18–24 were chosen for the study among current University of California, Santa Barbara students via major department mailing lists. Participants were contacted regarding the procedure of the study and asked to complete a consent form before receiving any other information. All subjects were also asked to complete an assessment form prior to enrollment in the study to verify any chronic medical conditions. None of the participants had listed any chronic medical conditions. Before the start of data collection, participants were briefed on the purpose and process to completing the study. Participants were seated in front of the computer in a padded chair and connected to the ear clip pulse sensor. The pulse sensor was attached to either the right or left earlobe. Participants were given a visual breath pacer described above and guided to breathe according to the instructions on the computer screen with a timer. For the first 2 min of the session, instructions asked the participants to follow the breath pacer. For the next 1 min, instructions asked the participants to breathe at their own pace. Such a pattern repeated for the whole duration of a 12 min session. Timer on the screen showed how much time was left for each part of the session as well as for the whole session. Change of the instructions on the screen was accompanied by a sound notification to make sure that the participants change their breathing pace at the correct times. Once the session was over, participants were given $5 in cash for compensation.

The data that were collected in the study consisted of data points that included magnitude and phase of pulse rate variability, pulse rate values, digital pulse signal, and corresponding times relative to the start of the session (trial). Data points were collected at a rate of 1 data point per 15 milliseconds. The magnitude and phase, however, were only computed once per breath cycle (10 s). We also collected binary data that told in which region of the session the subject was or was not following the breath pacer.

Once the data were collected for every session, it was rescaled such that a single data point corresponded to a single breath. The primary metric was the magnitude of the pulse rate variability. For each data point (breath) we computed the average and the standard deviation of the magnitude over the previous 4 breaths. We then introduced 3 metrics (each for a given breath): change of magnitude with respect to average over previous 4 breaths excluding current; ratio of change of magnitude with respect to average over previous 4 breaths excluding current to the standard deviation computed over the same interval; ratio of change of magnitude with respect to average over previous 4 breaths including current to the standard deviation computed over the same interval.

Using these metrics, we explored two possible models to detect non-compliance with the breath pacer. In the first technique, we trained a linear regression model with Python scikit-learn library, where the output was trained based on collected binary data of subjects either being compliant with the breath pacer or not.

In the second technique (later referred as running standard deviation technique), we computed the ratio of change of magnitude with respect to average over previous 4 breaths excluding current to the standard deviation computed over the same interval; ratio of change of magnitude with respect to average over previous 4 breaths including current to the standard deviation computed over the same interval. If the former exceeded 1.8 and the latter exceeded 3, we assumed that the subject’s breath was not compliant with the breath pacer. These thresholds were determined experimentally to yield the best accuracy of the algorithm.

## Results

**Fig. 3:**
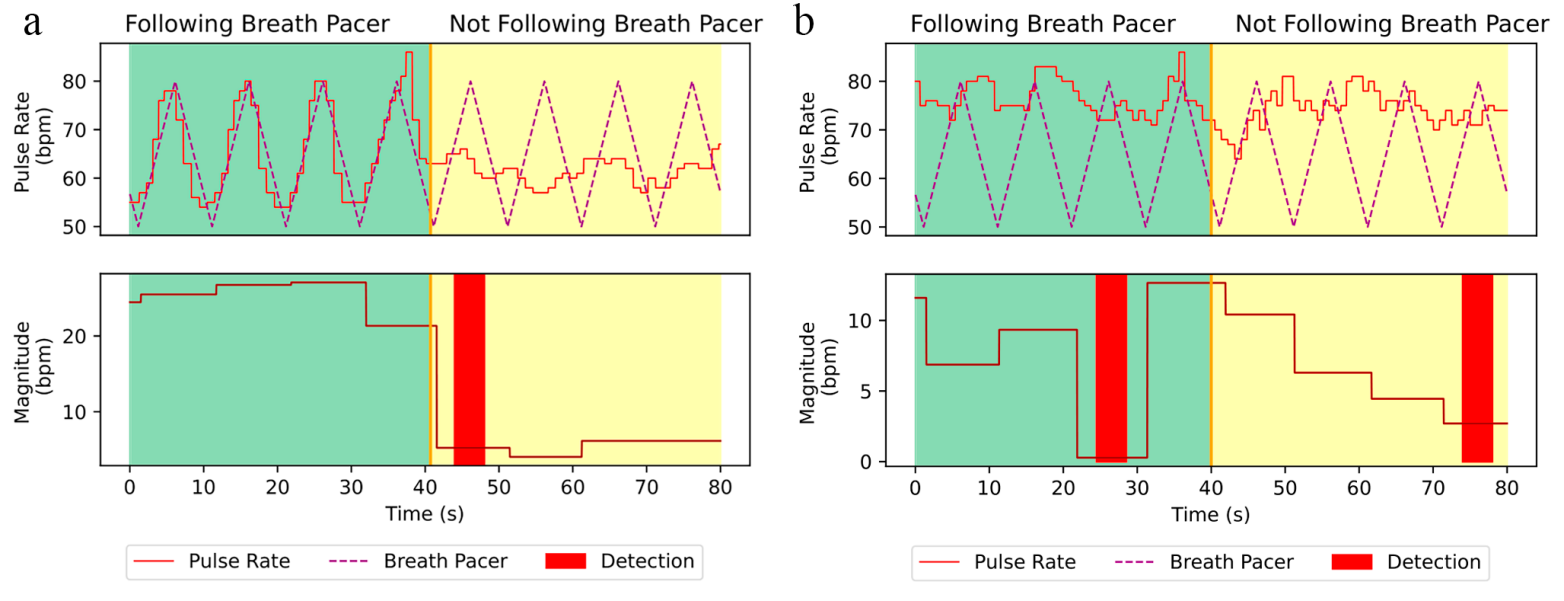
The magnitude of the pulse rate variability (PRV) that is in-phase with the breath pacer, also known as the magnitude of the Respiratory Sinus Arrhythmia (RSA). Subject with large PRV (**3a**): when the person stops following the breath pacer, the magnitude drops significantly which can be used to identify non-compliance with the breath pacer. Subject with low PRV (**3b**): magnitude does not correlate well with following or not following the breath pacer, which makes it hard to make a detection

We tested how well the models performed on the collected data. The key assumption was that the in-phase with breathing component of pulse rate variability was dropping significantly as the person stopped following the breath pacer and started breathing at his or her own rate (Fig. 3a). However, as was revealed during the study, this approach does not work for all people. For example, if a person has low pulse rate variability (Fig. 3b), then such a method cannot be used to reliably detect compliance with the breath pacer.

**Fig. 4:**
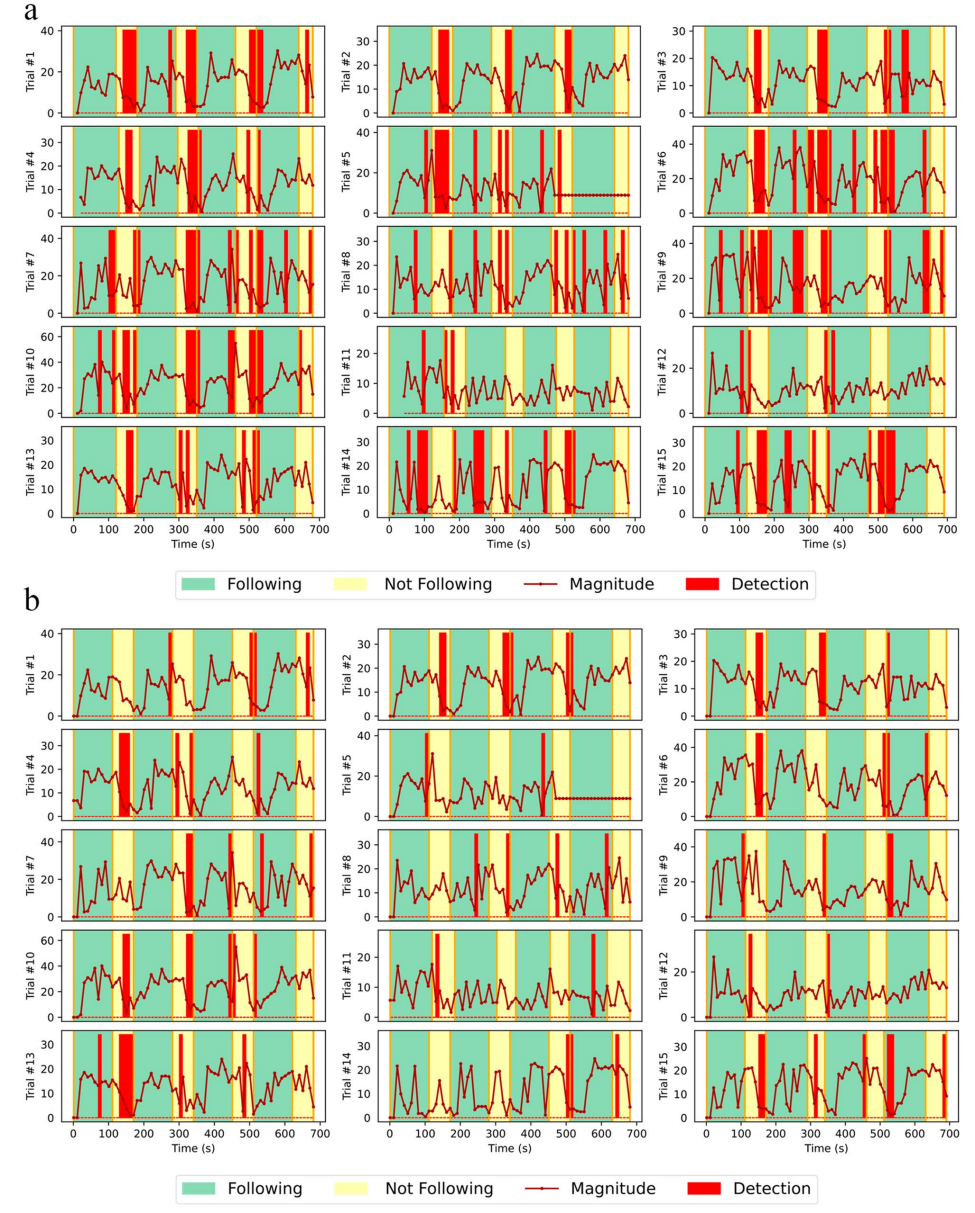
Detection of non-compliance with the breath pacer for different trials from the model based on logistic regression technique (**4a**) and running standard deviation technique (**4b**). Thicker detection bars indicate that the model detects non-compliance for several breaths in a row. Logistic regression model tended to have more false positive detections, while running standard deviation tended to have more false negative detections

**Fig. 5:**
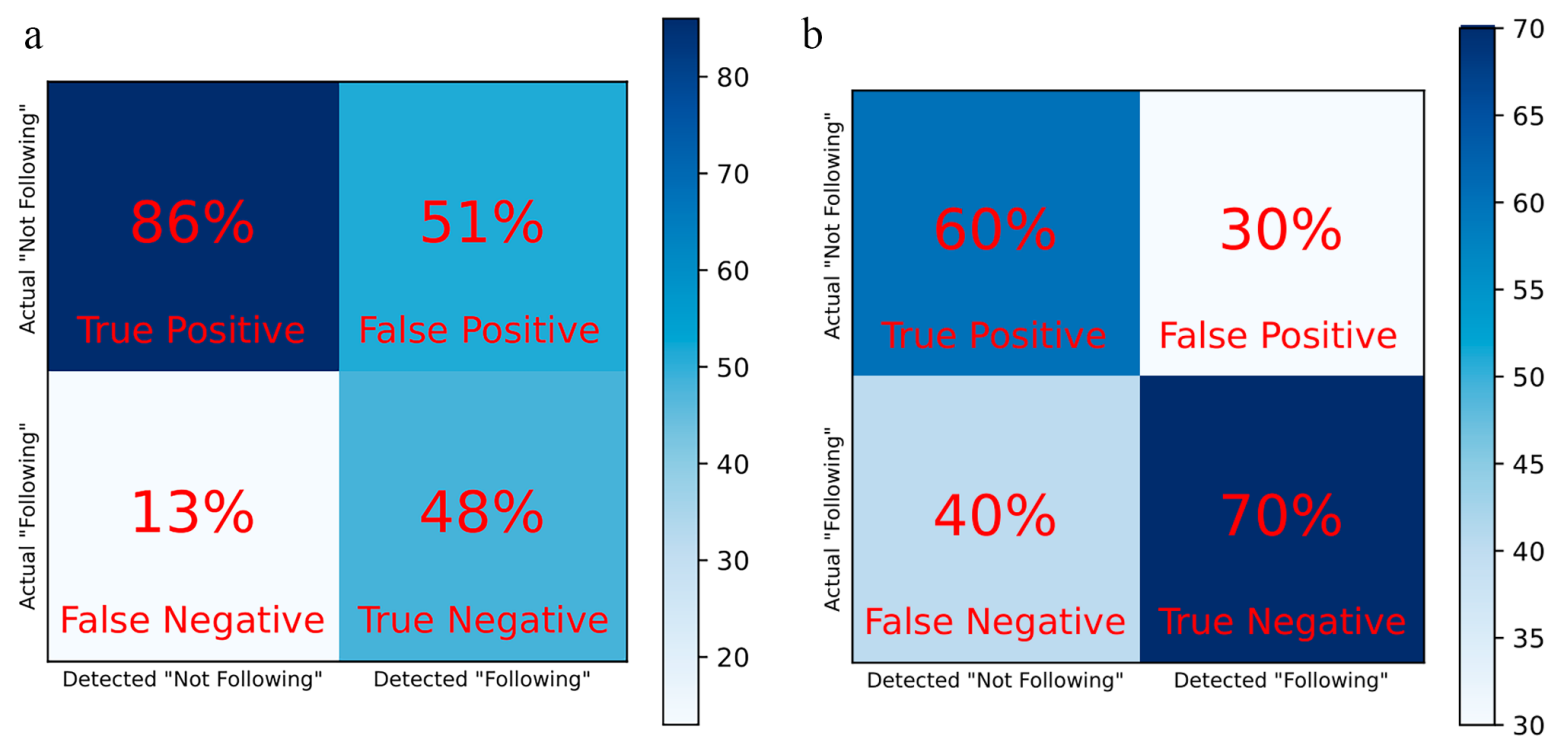
Confusion matrix for logistic regression technique (**5a**) and running standard deviation technique (**5b**). True positive is defined as correct detection of non-compliance, false positive is defined as incorrect detection of non-compliance, true negative is defined as correct detection of compliance, and false negative is incorrect detection of compliance

We present the performance of both logistic regression and running standard deviation (Fig. 4a and b correspondingly). Here the red bars denote the points in the session where an algorithm detects non-compliance with the breath pacer. The logistic regression model tended to have a lot of false positive detections, while running standard deviation tended to have more false negative detections. Quantitatively, we can describe each model with a confusion matrix (Fig. 5.) Here true positive is defined as correct detection of non-compliance, false positive is defined as incorrect detection of non-compliance, true negative is defined as correct detection compliance, and false negative is incorrect detection of compliance. From the confusion matrices we can compute an accuracy, defined as (True Positive + True Negative)/(True Positive + True Negative + False Positive + False Negative) (Fig. 5), for each model. The values are 67% for logistic regression technique, and 65% for running standard deviation technique.

**Fig. 6:**
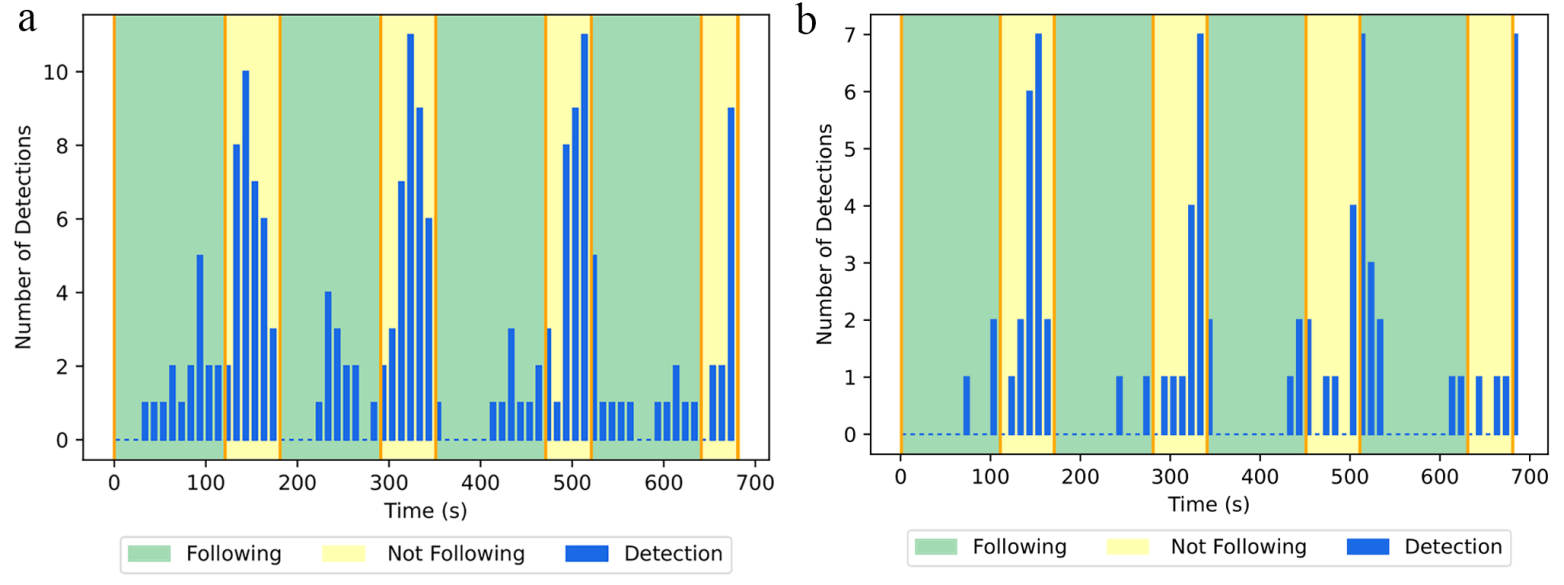
Cumulative distribution of non-compliance detections for logistic regression technique (**6a**) and running standard deviation technique (**6b**)

Here we also present the cumulative distribution of the device detections over the whole session for all subjects. Overall, we can see that both logistic regression (Fig. 6a) and running standard deviation (Fig. 6b) exhibit a trend and cluster around regions of non-compliance with the breath pacer. However, quantitatively, it is difficult to use such data for detection of non-compliance with the breath pacer.

## Discussion

In this study we have collected and analyzed that data from 6 subjects, where each subject participated in 2–3 trials (sessions). Overall, we were able to identify that our assumption that pulse rate variability can be used in order to detect non-compliance with the breath pacer was not valid for the majority of subjects. Although the models performed relatively well for some (for example, trial #1 on Fig. 4a or trial #3 on Fig. 4b), both models failed for most of the subjects. Based on these results, we can confirm that existing methods can demonstrate much better results for the purpose of breath pacer compliance and breathing rate detection (Benetazzo et al., 2014; Nam et al., 2016a, b; Avalur, 2013; Hernandez et al., 2015; Shen et al., 2017; Prigent et al., 2021).

Our study contained several limitations that could potentially be eliminated in further research. One issue is that we were not controlling the true (e.g. actual) breathing rate of people. Here, we implicitly assumed that people were indeed following the breath pacer and were breathing at a specific frequency, although we have no real data to confirm it. In the future studies, we would like to use some independent metric such as a chest strap sensor to measure the true breathing rate of the subject.

## Conclusion

In conclusion, in this paper we presented the description of the study on exploring the possibility of using the pulse rate variability in order to monitor the compliance with the breath pacer for people during meditation sessions. The device that was used in the study collected data concerning the pulse rate and magnitude of pulse rate variability of subjects, and actual compliance of the participants with the breath pacer. Specific metrics were derived from these data in order to create an algorithm to detect compliance with a breath pacer using just pulse rate and pulse rate variability data. We constructed two techniques, one based on logistic regression, and the other based on running standard deviation. The accuracy of both models was around 67% and 65% respectively, suggesting that pulse rate variability might be not the best metric in order to monitor compliance with the breath pacer, and that existing methods involving visual, audio, and motion signals perform better (Benetazzo et al., 2014; Nam et al., 2016a, b; Avalur, 2013; Hernandez et al., 2015; Shen et al., 2017; Prigent et al., 2021). We have also highlighted the limitations of the study, particularly the assumption that subjects followed breath pacer accurately, which may not always hold true. In summary, this study provided valuable insights into the challenges and complexities of using pulse rate variability in order to detect compliance with the breath pacer, and suggested that existing methods perform significantly better for this purpose.
